# Photosynthetic acclimation of *Grammatophyllum speciosum* to growth irradiance under natural conditions in Singapore

**DOI:** 10.1186/s40529-017-0210-x

**Published:** 2017-12-05

**Authors:** Jie He, Regina M. P. Lim, Sabrina H. J. Dass, Tim W. Yam

**Affiliations:** 1 Natural Sciences and Science Education Academic Group, National Institute of Education, Nanyang Technological University, 1 Nanyang Walk, Singapore, 637 616 Singapore; 2 Singapore Botanic Gardens, National Parks Board, 1 Cluny Road, Singapore, 25956 Singapore

**Keywords:** CO_2_ assimilation, Photosynthetic pigments, F_v_/F_m_ ratio, Leaf temperature, Relative water content, Specific leaf area, Stomatal conductance, Total reduced nitrogen

## Abstract

*Grammatophyllum speciosum*, a native species to Singapore, have become extinct mainly due to habitat loss. Recently, Singapore has reintroduced *G. speciosum* into the natural environment under the orchid conservation programme. In this study, leaves of *G. speciosum* grown under low light (LL) under natural conditions had faster expansion rate and higher specific leaf area than leaves grown under intermediate light (IL) and high light (HL). All leaves had more than 95% midday relative water content. Although midday F_v_/F_m_ ratios were lower in HL leaves than in IL and LL leaves, none of them exhibited chronic photoinhibition. HL leaves had upregulated their light utilization through higher photochemical quantum yield (ΔF/F_m_′) and greater electron transport rate. HL leaves also had higher non-photochemical quenching, indicating that they had higher capability to dissipate excess light as heat, which was supported by their lower chlorophyll but higher carotenoids content. Although there was a linear correction between leaf temperature and photosynthetic photon flux density (PPFD), no correlations were found between stomatal conductance (g_s_) and PPFD, g_s_ and leaf temperature. Light-saturated photosynthetic CO_2_ assimilation rate (*A*_*sat*_) was significantly higher in HL leaves than those of IL and LL leaves. However, all leaves had similar light-saturated stomatal conductance. Although LL leaves had higher leaf total reduced nitrogen that those of IL and HL leaves, none of them seemed to suffer from nitrogen deficiency during the experimental period. To conclude, *G. speciosum* is able to survive under different growth irradiances without watering and adding fertilizers.

## Background

Singapore houses 226 species of native tropical orchids. However, many of our natural habitats have been destroyed due to urbanisation. Besides the five common species, 178 species are extinct while the rest are either critically endangered or vulnerable (Ng 1994). One of the extinct species is *Grammatophyllum speciosum*, also known as the tiger orchid, which is the largest epiphytic C_3_ orchid plant in the world. Since 1995, The National Parks Board (NParks) in Singapore has been efficacious in propagating and reintroducing *G. speciosum* in its orchid conservation programme (Yam et al. 2010). More than 80% of native orchids planted on trees and ground in parks and nature reserve areas under different growth irradiances have survived (Yam 2013; Yam et al. 2011). Those could not have suffered from the complex interactions of high irradiances, high temperature, drought stress and nutrient deficiency.

Under tropical field conditions, orchids cope with variation of light intensities daily. Excessive light is one of the environmental stresses experienced by orchid plants (He et al. 1998, 2004, 2014; Koh et al. 1997; Tay et al. 2015). When they absorb excess light energy, if not dissipated as heat, it can be detrimental to photosystem II (PSII), known as dynamic or chronic photoinhibition (He et al. 1996). Dynamic photoinhibition is a reversible downregulation mechanism to reduce the light utilisation efficiency by diverting the excessive energy to the xanthophyll cycle so as to protect PSII reaction centres from photodamage (Demmig-Adams and Adam III 1992; Chow 1994; Osmond 1994). On the other hand, the excess energy could cause damage to the photosynthetic reaction centres, particularly of PSII, leading to sustained photoinhibition and then reduction of plant growth and productivity (Powles 1984; Osmond 1994; Barber 1995; Adams III et al. 2006). Chronic photoinhibition is the slower reversible loss of the function of PSII which is dependent on the repair and recovery rates of D1 protein in PSII reaction centres (Osmond 1994). Long term exposure to high irradiance would lead to irreversible oxidation of the chlorophyll (Chl) and destruction of chloroplasts due to formation of reactive oxygen species (ROS) (Lichtenthaler and Wellburn 1983). Thus, orchids grown under different growth irradiances, have different amount of Chl a, b and carotenoids (Car) (Lichtenthaler and Burkart 1999) with plants under high irradiance having more Car especially xanthophyll to photoprotect PSII from damage.

When the absorbed light energy exceeds the requirement of carbon fixation for light energy under high temperature or water deficit condition, photoinhibition is exacerbated (Lawlor and Tezara 2009). With water deficit, there is a reduction in leaf turgor potential and water content (Gomes et al. 2008); formation of ROS, which destroys the cell membrane, nucleic acid and protein (Ashraf 2009) such as Rubisco, an important carboxylase in C_3_ plants, thus decreasing carboxylation efficiency. Also, stomata close with water deficit to reduce transpiration which affects the leaf temperature (Farquhar and Sharkey 1982). Stomatal closure could also reduce photosynthesis by direct effects on the photosynthetic capacity of the mesophyll cells (Jones 1998). Closure of stomata results in reduced light-saturated stomatal conductance (*g*_*s sat*_) and photosynthetic CO_2_ assimilation, (*A*_*sat*_) affecting leaf growth and productivity (He et al. 2001).

Photoinhibition can also be aggravated by the lack of nutrients. Nitrogen (N) is often considered as the most limiting nutrient for the growth and yield of plants worldwide (Angus et al. 1993). N is a key component for protein synthesis and specifically for the maintenance of the abundant proteins associated with the photosynthetic apparatus (Loebl et al. 2010). Since PSII reaction centers are constantly damaged by high irradiances, cells need N to synthesis new proteins required for PSII repair (Nishiyama et al. 2006). If PSII repair cannot counteract photoinactivation due to N depletion, then the cells are subjected to chronic photoinhibition (Ragni et al. 2008).

Very little is known about the growth and photosynthetic performances of *G. speciosum* grown under natural tropical conditions. Furthermore, leaf photosynthetic gas exchange of plants subjected to mild drought stress may be largely due to stomatal limitation, rather than biochemical factors such as Rubisco protein (Lawlor and Cornic 2002; Bota et al. 2004; Flexas et al. 2006). Since all *G. speciosum* plants grown under natural conditions used for this project were maintenance free without watering and fertilization, it was often questioned that if N deficiency had occurs in these plants. Thus, using the native orchid species*, G. speciosum*, this project aimed to study (1) the effects of different growth irradiances on leaf growth, water relations and photosynthetic gas exchanges, (2) photosynthetic utilization of light energy and, (3) the concentration of leaf total reduced nitrogen (N) from leaves grown under different growth irradiances.

## Methods

### Plant materials

*Grammatophyllum speciosum* plants were planted on the grounds of National Institute of Education, Singapore in October 2012 by NParks under different growth irradiances, namely high light (HL, open field), intermediate light (IL) and low light (LL) respectively. The IL and LL conditions were obtained from different levels of shading by the surrounding trees. The average maximal photosynthetic photon flux density (PPFD) during midday at the top of the canopy were *ca.* 2000, 1200 and 600 under HL, IL and LL, respectively on sunny days. About 30 plants were planted under each of the light conditions. The 5th fully expanded leaves from the top of the plants were tagged for measurements from January to November 2013. These orchids were neither watered nor fertilized.

### Measurement of leaf length and specific leaf area (SLA)

The lengths of the tagged leaves were measured thrice by inserting the stick abaxially. Six weeks later, leaves were harvested. To estimate SLA, six leaf discs (3.4 cm^2^) per individual condition were collected with a cork borer. The leaf’s midveins were avoided to reduce variations. The discs were dried to constant mass at 80 °C, and then weighed. Dry weight (DW) was used to calculate the SLA, the ratio of leaf area to DW. The measurements were carried out from February to March 2013.

### Measurement of midday leaf relative water content (RWC)

On sunny days during March and October, leaves were harvested during midday. Six small squares (1 cm by 1 cm) were cut from one leaf before weighing the fresh weight (FW) instantly with an analytical balance (Sartorius). The samples were floated on water in the dark for 24 h before measuring their saturated weight (SW). They were then dried in the oven at 80 °C for 72 h before DW was obtained. RWC was calculated as RWC = (FW − DW)/(SW − DW) × 100%.

### Measurements of photosynthetic photon flux density (PPFD), predawn and midday F_v_/F_m_ ratios

PPFDs were measured using a photosynthetically available radiation quantum sensor and reading unit (Skye Instruments Ltd, Llandrindod, UK). They were measured from six different positions above the leaves for HL, IL and LL respectively. Chl fluorescence F_v_/F_m_ ratios measured the potential efficiency of excitation energy captured by PSII. The non-destructive measurements were carried out predawn and during midday (1200–1300 h) with the Plant Efficiency Analyser, PEA (Hansatech Instruments Ltd, England) on sunny days in March and October. Attached leaves were pre-darkened with clips for 15 min before measurements. Dark-adapted leaves were placed under the light pipe and irradiated with the pulsed lower intensity-measuring beam to measure F_0_, basal Chl fluorescence. F_m_, maximum Chl fluorescence was assessed by 0.8 s of saturated pulse (> 6000 mol m^−2^ s^−1^). The variable fluorescence yield, F_v_, was determined by F_m_–F_o_. The efficiency of excitation energy captured by open PSII reaction centres in dark adapted leaves was estimated by the fluorescence F_v_/F_m_ ratio.

### Measurements of different Chl fluorescence parameters

These measurements were carried out in October. Newly fully expanded leaves were harvested at 0800 h for Chl fluorescence analysis. The effective photochemical quantum yield (ΔF/F_m_′), electron transport rate (ETR) and non-photochemical quenching (NPQ) of Chl fluorescence were determined using the Imaging PAM Chl Fluorometer (Walz, Effeltrich, Germany) at 25 °C in the laboratory. Leaf discs were pre-darkened for 15 min before the measurements. Images of fluorescence emission from the PAM Chl Fluorometer, were digitized within the camera via a Firewire interface (400 Mb/s) (Firewire-1394, Austin, TX, USA) to a personal computer for storage and analysis. The details of measuring light pulses, actinic illumination and saturation pulses were described previously (He et al. 2011). Rapid light curve measurements in the presence of actinic illuminations (Schreiber et al. 1997) were obtained through the application of a series of 10-s light exposures with increasing irradiance from 1 to 1585 µmol photons m^−2^ s^−1^. In the presence of actinic illumination, the current fluorescence yield (F_t_ = F), and the maximum fluorescence (F_m_′) at the steady state, were determined, from which the effective PSII quantum yield, ΔF/F_m_′ [(F_m_′ − F)/F_m_′)] and ETR (PPFD × ΔF/F_m_′ × 0.5 × 0.84) could be calculated. The use of two photons is necessary to transport one electron (factor 0.5). Correction factor 0.84 takes into account that only a fraction of incident light is really absorbed by photosynthesis. NPQ was defined as: NPQ = (F_m_ − F_m_′)/F_m_′ (Rascher et al. 2000).

### Determination of photosynthetic pigments

These measurements were carried out in both March and October. Newly fully expanded leaves were harvested at 0800 h. Samples of 0.05 g were weighed and cut into very small pieces before soaking in 5 ml of *N*,*N*-dimethylformamide in the dark for 48 h at 4 °C. They were then quantified spectrophometrically (Du 650, Beckman, USA) using the procedure of Wellburn (1994).

### Measurements of diurnal changes in g_s_ and leaf temperature

The *g*_s_ and leaf temperature were measured every 2 h from 0800 to 1800 h using the leaf porometer chamber (SC-1, Decagon, U.S.) with a fixed diffusion path to the leaf surface. These measurements were carried out in March on three sunny days.

### Measurements of *A*_*sat*_ and *g*_*s sat*_

These measurements were carried out in both March and October on three sunny days. They were measured simultaneously with an open infrared gas analysis system with a 6 cm^2^ chamber (LI-6400, Biosciences, US) between 0900 and 1030 h. Readings were measured with a LED light source, which supplied a saturated PPFD of 1000 mol m^−2^ s^−1^. The light source emitted light in the wavelength range of 660–675 nm. Average ambient [CO_2_] and relative humidity in the chamber were 400 ± 5 µmol mol^−1^ and 70%, respectively.

### Determination of leaf total reduced nitrogen (TRN)

In October, the same leaves used for the measurements of *A*_*sat*_ and *g*_*s sat*_ were harvested immediately after recording the values of *A*_*sat*_ and *g*_*s sat*_. The leaves were then used for the analysis of TRN. Leaf TRN concentration was determined by Kjeldahl digestion of dried samples in concentrated H_2_SO_4_. Dry samples of 0.15 were placed into a digestion tube with a Kjeldahl tablet and 2.5 ml of concentrated H_2_SO_4_. The mixture was then digested (about 90 min) until clear. After the digestion was completed, the mixture was allowed to cool for 30 min and the TRN was determined by with a Kjeltec auto 2300 analyser.

### Statistical analysis

One-way ANOVA was used to test for significant differences among different growth irradiances, using Tukey’s multiple comparison tests to discriminate the means (MINITAB, Inc., Release 15, 2007).

## Results

### Leaf length, SLA and midday leaf RWC

**Fig. 1:**
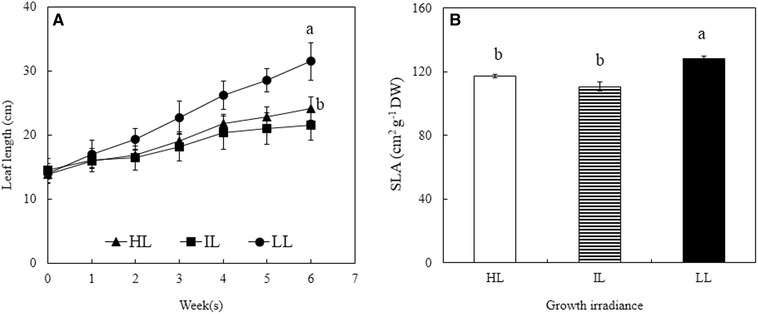
Leaf length (**A**) and SLA (**B**) of young expanding leaves of *G. speciosum* under different growth irradiances. Each value is the means from 6 different leaves of 6 different plants. Vertical bars represent SE. Means with same letter above the bars are not statistically different (p > 0.05) as determined by Tukey’s test

**Fig. 2:**
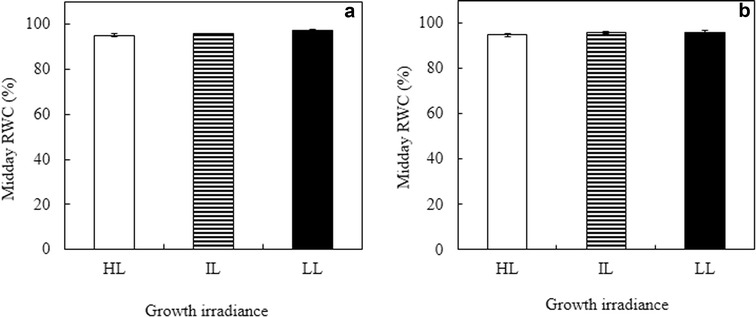
Midday RWC of young expanding (**a**) and fully expanded (**b**) leaves of *G. speciosum* under different growth irradiances. Each value is the mean from 6 different leaves of 6 different plants. Vertical bars represent SE

All leaves increased gradually in length over 6 weeks of measurement. Leaves of *G. speciosum* plants grown under LL seemed to grow faster than leaves grown under IL and HL, which were the longest after 6 weeks (Fig. 1A, p < 0.05). However, there was no significant difference in leaf length between IL and HL leaves after 6 weeks of expansion (Fig. 1A, p > 0.05). SLA was not significantly different between leaves grown under HL and IL but LL grown leaves had significantly higher SLA (Fig. 1B). No significant differences in midday leaf RWC were observed across the plants grown under three different growth irradiances (Fig. 2, p > 0.05). Results of midday leaf RWC shown in Fig. 2 were obtained on sunny days in October. Same measurements were carried out on sunny days in March and all leaves had midday RWC greater than 95%.

### Midday and predawn F_v_/F_m_ ratios measured under field conditions

**Fig. 3:**
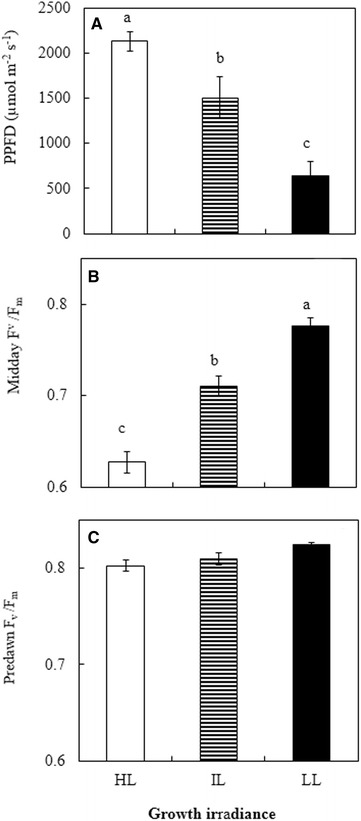
Midday PPFD (**A**), Midday (**B**) and predawn (**c**) F_v_/F_m_ ratios and of leaves of *G. speciosum* under different growth irradiances on sunny days. Each value is the mean from 6 different leaves of 6 different plants measured from three sunny days. Vertical bars represent standard errors. Means with same letter above the bars are not statistically different (p > 0.05) as determined by Tukey’s test

These measurements were carried out in both March and October on sunny days. Similar results were obtained and thus, only data from October are shown in Fig. 3. Among all leaves, the lowest midday F_v_/F_m_ ratio was recorded from the leaves that were grown under HL followed by those grown under IL and the highest F_v_/F_m_ ratio was found in leaves exposed to LL (Fig. 3B, p < 0.05). Differences in midday F_v_/F_m_ ratios corresponded with the levels of light to which they were exposed (Fig. 3A), that was, the higher the PPFD illuminated the leaves, the lower the midday F_v_/F_m_ ratio. However, there were no significant differences in predawn F_v_/F_m_ ratio and all the leaves had predawn F_v_/F_m_ ratio ≥ 0.8 (Fig. 3C).

### Photochemical efficiency and photosynthetic pigments measured in the laboratory

**Fig. 4:**
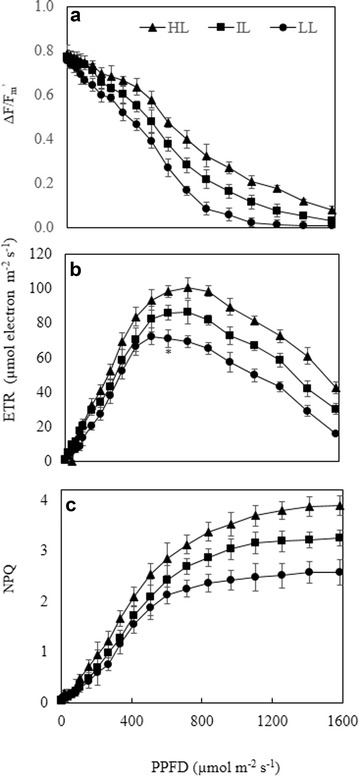
ΔF/F_m_′ ratio (**a**), ETR (**b**) and NPQ (**c**) of leaves of *G. speciosum* grown under different growth irradiances. Each value is the mean from 30 readings of 6 different leaves from 6 different plants. Vertical bars represent standard errors. When the standard error bars cannot be seen, they are smaller than the symbols

Parameters such as F/F_m_′ (photochemical quantum yield at actinic light) ETR and NPQ were used to explore the utilization of light energy by leaves grown under different growth irradiances. All results shown in Fig. 4 were obtained in October. The light response curves of these parameters were measured under different PPFDs in the laboratory from 1 to 1585 µmol m^−2^ s^−1^. When measured at the lowest PPFD of 1 µmol m^−2^ s^−1^, ΔF/F_m_′ values were not significantly different among the different leaves ranging from 0.788 to 0.754. However, the values of ΔF/F_m_′ decreased to 0.637, 0.549 and 0.468 measured at PPFD of 415 µmol m^−2^ s^−1^ for leaves grown under HL, IL and LL, respectively (Fig. 4a). The differences in ΔF/F_m_′ among the different leaves at this PPFD were significantly different (p < 0.05). A further decreases of ΔF/F_m_′ in all leaves were observed when PPFD was higher than 415 µmol m^−2^ s^−1^ with their value almost zero at PPFD of 1585 µmol m^−2^ s^−1^. However, the rate of deceases in ΔF/F_m_′ was the fastest in leaves grown under LL followed by that of leaves under IL with increasing PPFD. Leaves grown under HL exhibited the slowest decrease rate of ΔF/F_m_′ with increasing PPF. The HL leaves had the highest values of ΔF/F_m_′ measured at higher PPFDs followed by the IL leaves and the LL leaves had the lowest values at each PPFD from 415 to 1255 µmol m^−2^ s^−1^ (Fig. 4a, p < 0.05).

Initially, there were steep increases of ETR values in all leaves with increasing PPFD until 715 µmol m^−2^ s^−1^, after which they all decreased sharply (Fig. 4b). Significant differences in ETR values were obtained among leaves grown under different grown irradiances from PPFD of 605 to 1585 µmol m^−2^ s^−1^ (p < 0.05). Although the changes of ETR to increasing PPFD were similar in all leaves, the ETR of LL leaves was the lowest at any given PPFD followed by that of IL leaves. The HL leaves had the highest ETR compared to those of IL and LL from 605 to 1585 µmol m^−2^ s^−1^.

There were gradual increases in NPQ of IL and HL leaves from 1 to 835 µmol photon m^−2^ s^−1^ after which they plateau around 2.69 and 3.12, respectively (Fig. 3C). The increase of NPQ was faster in HL leaves than in IL at the any given PPFD from 715 to 1585 µmol photon m^−2^ s^−1^ (p < 0.05). For LL leaves, although its NPQ also increased with increasing PPFD, it plateaus at much lower PPFD of 605 µmol photon m^−2^ s^−1^ compared to those of IL and HL leaves. At each given PPFD, LL leaves had the significantly lowest NPQ that those of IL and HL leaves from PPFD of 715 µmol photon m^−2^ s^−1^ onwards (p < 0.05).

**Fig. 5:**
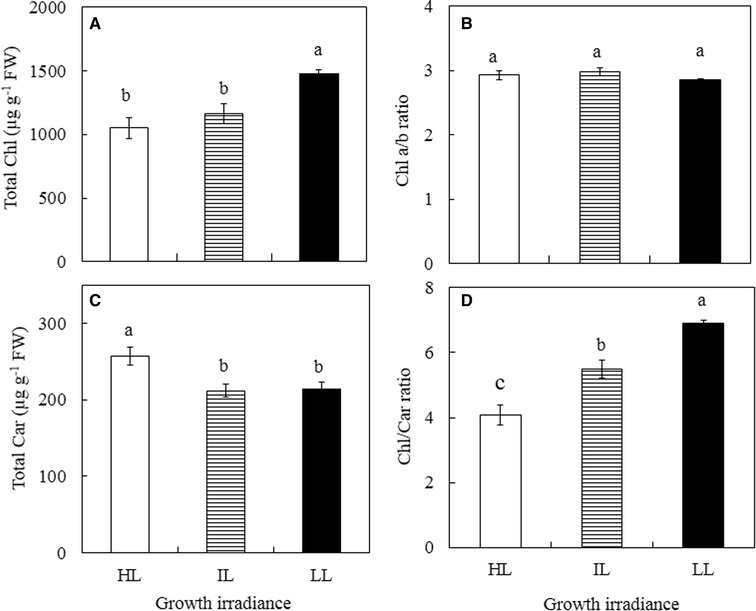
Total Chl content (**A**), Chl a/b ratio (**B**), total Car content (**C**) and Chl/Car ratio (**D**) of leaves of *G. speciosum* grown under different growth irradiances. Each value is the mean from 4 different leaves of 4 different plants. Vertical bars represent standard errors. Means with same letter above the bars are not statistically different (*p* > 0.05) as determined by Tukey’s test

Photosynthetic pigments of leaves of *G. speciosum* grown under different growth irradiances were analyzed in both March and October. Similar trends were observed and thus, only results obtained from Oct are presented in Fig. 5. The total Chl content was significantly higher in LL leaves than those of IL and HL leaves (Fig. 5A, p < 0.05). However, there was no significant difference in Chl a/b ratio across the different growth irradiances (Fig. 5B, p > 0.05). Total Car content of LL and IL leaves was not significantly different (p > 0.05) but they were much lower than that of HL leaves (Fig. 5C, p < 0.05). Chl/Car ratios of LL and IL leaves were higher than that of HL (Fig. 5D, p < 0.05) due to their higher Chl content (Fig. 5A) or lower Car content (Fig. 5C).

### Diurnal changes of PPFD, g_s_ and leaf temperature and correlations among them

**Fig. 6:**
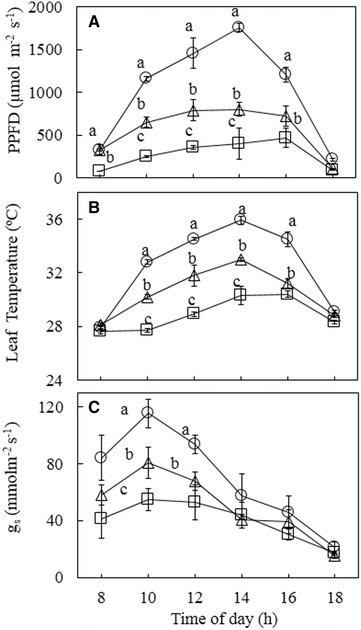
Diurnal changes in PPFD (**A**), leaf temperature (**B**) and g_s_ (**C**) of leaves of *G. speciosum* grown under different growth irradiances. Each value is the mean from 6 different leaves of 6 different plants. Vertical bars represent standard errors. Means with same letter are not statistically different (p > 0.05) as determined by Tukey’s test

**Fig. 7:**
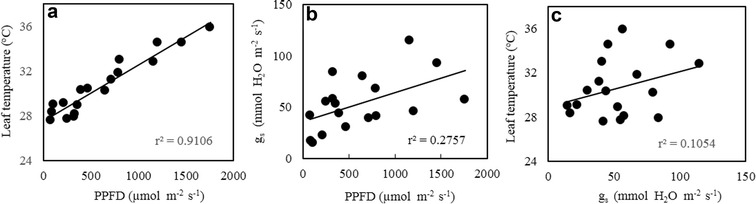
Correlations between leaf temperature and PPFD (**a**), g_s_ and PPFD (**b**), leaf temperature and g_s_ (**c**) of leaves of *G. speciosum* grown under different growth irradiances. All data derived from Fig. 6

These measurements were carried out in March on three sunny days. Similar results were obtained among the three different sunny days. The values of PPFD (Fig. 6A) and leaf temperature (Fig. 6B) increased parallel from 0800 to 1400 h and then decreased after that. There were significant differences in PPFD (Fig. 6A), g_s_ leaf temperature (Fig. 6B) and g_s_ (Fig. 6C) across the different growth irradiances at 1000 h, when HL leaves had the highest values followed by those of IL leaves (p < 0.05). The LL leaves had the lowest leaf temperature and g_s_ at the same given time compared to those of IL and HL leaves (p < 0.05). It was interesting to see that g_s_ decreased from 1000 h onwards in all leaves. A close linear correlation between leaf temperature PPFD was established in leaves grown under different growth irradiance (Fig. 7a, r^2^ = 0.9106). However, there were no correlations between *g*_s_ and PPFD (Fig. 7b, r^2^ = 0.2757), leaf temperature and *g*_s_ (Fig. 7c, r^2^ = 0.1054).

### *A*_*sat*_, *g*_*s sat*_ and Leaf TRN

**Fig. 8:**
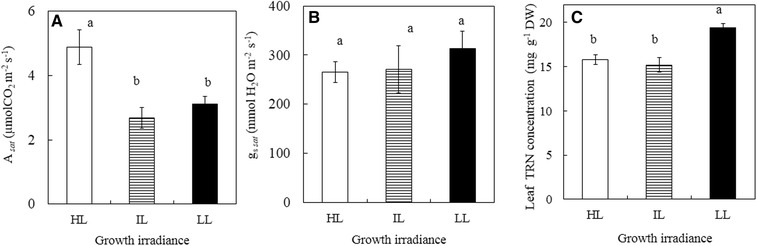
*A*_*sat*_ (**A**), g_s *sat*_ (**B**) and TRN (**C**) of leaves of *G. speciosum* grown under different growth irradiances. Each value is the mean from 4 different leaves of 4 different plants. Vertical bars represent standard errors. Means with same letter above the bars are not statistically different (p > 0.05) as determined by Tukey’s test

*A*_*sat*_ of HL leaves was significantly higher than those of IL and LL leaves but not significantly different between IL and LL leaves (Fig. 8A, p < 0.05). However, there was no significant difference in the g_s *sat*_ among all leaves (Fig. 8B, p > 0.05). For the leaf TRN concentration, there was no significant difference between HL and IL leaves (Fig. 8C, p > 0.05) but LL leaves had significant higher leaf TNR concentration compared to those of HL and IL leaves (Fig. 8C; p < 0.05). All results shown in Fig. 8 were obtained in October. *A*_*sat*_ and *g*_*s sat*_ were also measured in March and the trends were similar to those presented in Fig. 8A, B.

## Discussion

The light environments for different leaves on the same plants vary according to the position of the sun and variable cloud cover. Furthermore, every leaf on the same plant was in different light environment due to its position on the plant in relation to the other leaves. These factors explained the differences in the variation in leaf development on the same plant. The extension zone of the *G. speciosum*, being a monocot, was enclosed by older leaves sheath shielding it from light (Dale 1988). This could be an added constraint. There might also be a wide variation in growth rate for leaves on the same plant. Too much light can cause detrimental damages to the photosynthetic apparatus (Lambers et al. 2008). On the other hand, insufficient light could reduce photosynthetic rates and a subsequent reduction in overall plant growth (He et al. 1998; Koh et al. 1997; He and Teo 2007). In the present study, leaves grown under LL seemed to expand significantly faster (Fig. 1A) and thinner reflected by higher SLA (Fig. 1B) than leaves grown under IL and HL. Under shady conditions, to optimise photosynthetic efficiency, SLA of leaves increased, resulting in longer and thinner leaves (Dale 1988). For plants grown under HL and IL conditions, reducing the surface area of leaves, leading to lesser water loss by evapotranspiration could be a strategy for water conservation (Tay et al. 2015).

Water deficit is one of the greatest limitations to the growth of epiphytic orchids (Laube and Zotz 2003; Zotz et al. 2010). However, in the present study, the midday RWC of all leaves grown under three growth irradiances were more than 95% for both young expanding (Fig. 2a) and fully expanded (Fig. 2b) leaves indicating that this orchid species did not suffer drought stress (Lizana et al. 2006; Lugojan and Ciulca 2011). Even though they were not irrigated, they could have a large supply of water from their pseudobulbs (Arditti 1992; He et al. 2011, 2014; Tay et al. 2015). Moreover, the study was carried out during the Northeast monsoon (October to November). The fat egg-shaped pseudobulbs of this tropical orchid store large amount of water during rainy season. Similar measurements were also carried out during the dry season (February to March). All leaves had more 95% midday RWC regardless of growth irradiances (data not shown). Stomatal regulation is one of the strategies preventing water loss (Yordanov et al. 2000). According to Cornic and Fresneau (2000), up to leaf RWC of about 70%, it is likely that stomatal closure plays the main role in preventing water loss (He et al. 2014; Tay et al. 2015). In the present study, decreased *g*_*s*_ from 1000 h onwards (Fig. 6C) supported this postulation.

In this study, on sunny days, leaves grown under HL had much lower midday F_v_/F_m_ ratio than those under IL and LL (Fig. 3B). HL leaves, being in open field, were exposed to maximum PPFD of 2133 μmol m^−2^ s^−1^, which were much higher than those of IL and LL leaves (Fig. 3A). The lowest midday F_v_/F_m_ ratio due to higher PPFD indicated that dynamic PSII photoinhibition was much more severe in HL leaves than in IL and LL leaves. Photoinhibition could even occur at moderate or low light when other adverse conditions such as high temperature is present (He et al. 1996, 1998; He and Teo 2007). Leaf temperature (Fig. 6B) increased parallel with PPFD (Fig. 3A) from 0800 to 1400 h and the close linear correlation between leaf temperature and PPFD (Fig. 7a), suggested that dynamic PSII photoinhibition under tropical natural conditions resulted from the combination of high PPFD and high leaf temperature (He et al. 1996, 1998). It was reported that a large supply of water allowed leaves under all growth irradiances to modulate their leaf temperature, preventing them from overheating achieved through the evaporation of water from stomata known transpiration (He et al. 2001; Mohotti and Lawlor 2002; Hetherington and Woodward 2003; Crawford et al. 2012) and thus, protecting their PSII from photoinhibitory damages (Jagtap et al. 1998; He et al. 2001). However, in the present study, *g*_*s*_ (Fig. 6C) increased from 0800 to 1000 h with increasing PPFD (Fig. 6A) in all leaves and it did not increase further with increasing PPFD and leaf temperature from 1000 h onwards. This result implied that *G. speciosum* had developed the avoidance of drought stress by widely opening stomata only for a short period of time in the early morning to conserve water (Ort 2001). Thus, the results of the present study did not support strategy of transpiration cooling as stomata of all leaves were partially closed with decreased g_s_ (Fig. 6C) under high temperatures during midday (Fig. 6B). Although there were no correlations between the stomatal conductance and PPFD (Fig. 7b); or leaf temperature (Fig. 7c); across the different irradiances and across the whole day, *g*_*s*_ (Fig. 6C) increased from 0800 to 1000 h with increasing PPFD (Fig. 6A). Stomata remain closed in the afternoon and opening only in the morning may play the main role in preventing water loss for this epiphytic tropical orchid (He et al. 2014; Tay et al. 2015). Furthermore, other environmental conditions such as humidity (Grantz 1990; Peak and Mott 2011) and vapour pressure deficit (VPD) (Day 2000; Shirke and Pathre 2004) may play crucial roles in regulating stomatal conductance.

Stomatal closure during midday could result in potential depletion of internal CO_2_. Low availability of CO_2_ at high light intensities may lead to the decrease of photosynthetic electron consumption, causing a dynamic photoinhibition (Osmond 1994; Cornic and Fresneau 2000). In the present study, all leaves had experienced dynamic photoinhibition measured by midday F_v_/F_m_ ratios that were below 0.8 (Fig. 3B). However, all leaves completely recovered through the night from dynamic photoinhibition as they all had predawn F_v_/F_m_ ratios greater than 0.8 (Osmond 1994). Rapidly reversible decreases in maximal PS II efficiency (i.e. lowered F_v_/F_m_ ratios of pre-darkened leaves) is a photoprotective energy dissipation process, even though rates of photosynthetic electron transport remain maximal and photosynthesis is not inhibited (Adams et al. 1999). In the present study, leaves grown under HL had greater dynamic photoinhibition, these leaves, however, had higher ΔF/F_m_′ (Fig. 4a), ETR (Fig. 4b), and NPQ (Fig. 4c), compared to those grown under IL and LL exhibiting their higher capacities of utilizing and dissipating light energy (He and Lee 2004; He et al. 2014). When a proportionally greater amount of the absorbed light cannot be utilized in photochemical activity at higher light levels, leaves normally upgregulated their Car level to dissipate excess photons through the xanthophyll cycle (Adams and Demmig-Adams, 1992; Demmig-Adams and Adams III 1992, 2006). When leaves grown under HL, there was an accompanied increase in Car (Fig. 5C), decrease in Chl (Fig. 5A) and thus, higher ratio of Car/Chl (Fig. 5D) to offer photoprotection (Armstrong and Hearst 1996; Puthur 2005). Shading increased Chl content of LL grown leaves (Fig. 5A, Anderson et al. 1991; Newman and Follett 1998). Plants adapted to HL are well known to have high Chl a/b ratio (Anderson 1986; Anderson and Osmond 1987). However, no significant differences were observed in Chl a/b ratios among all leaves. The low Chl content in leaves of HL plants exposing to maximal PPFD above 1500 μmol m^−2^ s^−1^ could be a result of adaptation (Anderson 1986).

Photosynthesis of healthy leaves increases proportionally to the increases in PPFD until its rate begins to saturate. Increases of photosynthetic CO_2_ assimilation rate with increasing PPFD for *G. speciosum* grown under all light conditions were very slow and they all began to show saturation under a PPFD about 600 µmol photon m^−2^ s^−1^ (data not shown). Using tropical crop species, Da Matta and colleagues (2001) studied the actual photosynthetic rate (*A*). They reported that *A*, determined under non-limiting light at ambient temperature and CO_2_, varied from 5.0 up to 26.3 µmol CO_2_ m^−2^ s^−1^. It was also reported previously, on an area basis, *A* covers a wide range, from less than 2 up to 70 µmol CO_2_ m^−2^ s^−1^ (Larcher 1995) and may even higher with a value of 80 µmol CO_2_ m^−2^ s^−1^ from *Amaranthus retroflexus* (Pearcy and Ehleringer 1984). In the present study, *A*_*sat*_ (Fig. 8A) was higher in leaves grown under HL than under IL and LL from *G. speciosum*, a tropical native C_3_ orchid species. Higher leaf photosynthetic rate normally parallels its higher stomatal conductance at either high or low VPD environments (Shirke and Pathre 2004). In this study, *g*_*s sat*_ did not seem to increase with increasing growth irradiance (Fig. 8B). VPD could affect the internal CO_2_ concentration (*C*_*i*_) however, all leaves had similar *C*_*i*_ with average values around 280–300 mol CO_2_ mol^−1^ between 0900 and 1030 h (data not shown). *A*_*sat*_ of 5 µmol CO_2_ m^−2^ s^−1^ (Fig. 8A) from HL grown *G. speciosum* leaves was much lower than most tropical C_3_ plants. The differences in *A* among species depend on the proportion of the absorbed excitation energy that is used for photosynthesis (Adams and Demmig-Adams 1992; Adams et al. 2002; Demmig-Adams and Adams 1992). On other hand, N availability is a major factor limiting growth and development of plants (Kraiser et al. 2011). It is a key component for protein synthesis and for the maintenance of the abundant proteins associated with the photosynthetic apparatus (Loebl et al. 2010). Although the LL grown leaves had higher total leaf TRN compared to HL and IL plants (Fig. 8C), none of them was suffered from N deficiency as all plants that had more than 1.5% N without supplying fertilizers to plants, during the experiment period (12 months after transplanting). Lower *A*_*sat*_ was not caused by degradation of Rubisco either as all leaves had similar levels of Rubisco protein (data not show). Based on the above discussion, low photosynthetic rate and slow growth of *G. speciosum* could be mainly due to the limitation of internal CO_2_ concentration resulting from closing stomata for a long period of time during the day to conserve water (Ort 2001).

## Conclusion

Leaves grown under LL expanded faster and they were thinner than those grown under IL and HL. Water deficit was not observed in any leaves as they could avoid drought stress by opening their stomata only during early morning and closing or partially closing them from midday onwards. Although HL grown leaves experienced dynamic photoinhibition but not chronic photoinhibition as these leaves had higher capacities of utilizing and dissipating light energy. Low photosynthetic rate of all leaves of *G. speciosum* was mainly due to the limitation of internal CO_2_ concentration instead of N deficiency. Slow but healthy growth of all *G. speciosum* under natural conditions was due to their different physiological acclimations to different growth environments.

## Abbreviations

A_sat_: light-saturated photosynthetic CO_2_ assimilation
Car: carotenoids
Chl: chlorophyll
DW: dry weigh
ETR: electron transport rate
F_o_: minimal fluorescence yield of a “dark-adapted” sample
F_m_ and F_v_: maximal and variable fluorescence yields obtained from a dark-adapted sample upon application of a saturation pulse of radiation, respectively
F_m_′: the maximum fluorescence at the steady state
F_t_: the current fluorescence yield
F/F_m_′: the effective photochemical quantum yield
F_v_/F_m_: ratio to estimate the maximum portion of absorbed quanta used in PSII reaction centers
FW: fresh weight
HL: high light
IL: intermediate light
LL: low light
*g*_*s*_: stomatal conductance
*g*_*s sat*_: light-saturated stomatal conductance
NPQ: non-photochemical quenching
PPFD: photosynthetic photon flux density
PS II: photosystem II
SLA: specific leaf area
RWC: relative water content
TRN: total reduced nitrogen
